# The Role of Ca^2 +^ in Maturation and Reprogramming of Bovine Oocytes: A System Study of Low-Calcium Model

**DOI:** 10.3389/fcell.2021.746237

**Published:** 2021-10-26

**Authors:** Lin Meng, Hongmei Hu, Zhiqiang Liu, Luyao Zhang, Qingrui Zhuan, Xue Li, Xiangwei Fu, Shien Zhu, Yunpeng Hou

**Affiliations:** ^1^State Key Laboratory of Agrobiotechnology, College of Biological Sciences, China Agricultural University, Beijing, China; ^2^Hubei Key Laboratory of Natural Medicinal Chemistry and Resource Evaluation, School of Pharmacy, Tongji Medical College, Huazhong University of Science and Technology, Wuhan, China; ^3^Key Laboratory of Animal Genetics, Breeding and Reproduction, College of Animal Science and Technology, China Agricultural University, Beijing, China

**Keywords:** Ca^2+^, bovine oocyte, maturation, reprogramming, ID1

## Abstract

[Ca^2+^]_i_ is essential for mammalian oocyte maturation and early embryonic development, as those processes are Ca^2+^ dependent. In the present study, we investigated the effect of [Ca^2+^]_i_ on *in vitro* maturation and reprogramming of oocytes in a lower calcium model of oocyte at metaphase II (MII) stage, which was established by adding cell-permeant Ca^2+^ chelator BAPTA-AM to the maturation medium. Results showed that the extrusion of the first polar body (PB1) was delayed, and oocyte cytoplasmic maturation, including mitochondrial and endoplasmic reticulum distribution, was impaired in lower calcium model. The low-calcium-model oocytes presented a poor developmental phenotype of somatic cell nuclear transfer (SCNT) embryos at the beginning of activation of zygotic genome. At the same time, oxidative stress and apoptosis were observed in the low-calcium-model oocytes; subsequently, an RNA-seq analysis of the lower-calcium-model oocytes screened 24 genes responsible for the poor oocyte reprogramming, and six genes (ID1, SOX2, DPPA3, ASF1A, MSL3, and KDM6B) were identified by quantitative PCR. Analyzing the expression of these genes is helpful to elucidate the mechanisms of [Ca^2+^]_i_ regulating oocyte reprogramming. The most significant difference gene in this enriched item was ID1. Our results showed that the low calcium might give rise to oxidative stress and apoptosis, resulting in impaired maturation of bovine oocytes and possibly affecting subsequent reprogramming ability through the reduction of ID1.

## Introduction

Somatic cell nuclear transfer (SCNT) is of much current interest with potential applications in the protection of endangered species, agriculture, and regenerative medicine (Wu and Hochedlinger, 2011; Tachibana et al., 2013; Iqbal et al., 2021; Son et al., 2021). So far, it has successfully produced offspring in pigs, monkeys, goat, and other animals (Hajian et al., 2020; Ouyang et al., 2021; Sun et al., 2021). SCNT can reprogram terminally differentiated somatic cells to the totipotent state when transplanted into enucleated mature oocytes (metaphase II, MII) (Gonzalez-Munoz and Cibelli, 2018), but its mechanisms remain unclear (Keefer, 2015). In oocytes, somatic nucleus is rapidly reprogrammed by cytosolic factors to gain pluripotency in a deterministic manner (Brambrink et al., 2006; Khaw et al., 2015) through a series of sequential events including protein exchange between the donor nucleus and ooplasm, chromatin remodeling, and pluripotency gene reactivation (Jullien et al., 2014; Wen et al., 2014); factors that influence the expression of maternal “reprogramming factor” in mature oocytes that is responsible for this reprogramming process remain largely unidentified, and understanding how the mature oocytes obtain a reprogramming ability is the key for improving the reprogramming procedure. A previous work has indicated that maternal reprogramming factor from MII oocytes is the most important factor for SCNT success.

Metaphase II oocytes are the receptor cells of SCNT. The quality of oocytes is directly related to the reprogramming ability of animals, which is an important factor affecting the efficiency of animal cloning and *in vitro* fertilization (An et al., 2019). In mammals, after germinal vesicle breakdown (GVBD), oocytes resume meiosis and undergo a maturation process including the stages of metaphase I (MI), anaphase I (AI), and telophase I (T1) up to MII. Then, meiosis is again arrested until fertilization occurs (Petr et al., 1999). As a ubiquitous intracellular messenger, Ca^2+^ changes in oocytes play an important role in the regulation of oocyte maturation, activation of oocyte fertilization, and early embryonic development (Whitaker, 2006; Gómez-Fernández et al., 2012; Shibahara et al., 2021). In addition, there are studies that show that the transient calcium oscillation induced by repetitive ionomycin or the treatment of calcium ionophore with strontium can increase the developmental capacity of SCNT embryos (Kim et al., 2012; Choi et al., 2013). Therefore, Ca^2+^ may regulate the reprogramming ability of oocytes in some way, which can be further verified by intervention of Ca^2+^ in oocytes. BAPTA-AM is an osmotic calcium chelator widely used in oocytes, which rapidly reduces cytoplasmic calcium (Ruddock et al., 2001; Dell’Aquila et al., 2002; Wang et al., 2017). Studies have shown that BAPTA-AM can regulate the fertilization and developmental ability of vitrified bovine oocytes and improve the survival rate and *in vitro* maturation (IVM) rate of porcine vitrified GV oocytes, so it can be used to explore the effects of Ca^2+^ on bovine oocyte maturation and SCNT embryo development.

It is well known that there are marked changes in the distribution of the mitochondria and endoplasmic reticulum during oocyte maturation, which supply the majority of intracellular energy and calcium oscillations for subsequent events such as fertilization (Cohen et al., 1998; Darbandi et al., 2017). Important cytoplasmic organelle distribution of human oocytes during maturation has been reported, and the most prominent feature was the gradual formation of heterologous complexes composed of variable elements of the endoplasmic reticulum and multiple mitochondria with primitive morphology (Trebichalská et al., 2021). After research investigation, we know that cytoplasmic damages that contain early apoptosis, the increased levels of reactive oxygen species (ROS), DNA damage, and so on, can block the development of oocytes (Zhang et al., 2019). Epigenetic reprogramming also occurs in SCNT including DNA methylation, histone modifications, etc., (Zhang et al., 2021). DMNT3A and DMNT3B catalyze *de novo* methylation, which is essential for establishing DNA methylation during development (Hsieh, 1999; Okano et al., 1999; Gouveia et al., 2020).

In the present study, we constructed a low-calcium model of oocytes to explore whether [Ca^2+^]_i_ controls the accumulated expression of maternal reprogramming factor in MII oocytes and whether it may play a special role in the process of somatic cell reprogramming. Results showed that [Ca^2+^]i controls the accumulated expression of maternal reprogramming factor and many key pluripotency genes in MII oocytes during IVM. [Ca^2+^]_i_ is also critical for oocyte maturation and subsequent SCNT embryo development. Considering that developmental defects of SCNT embryos under lower calcium model first appear at the time of zygotic genome activation (ZGA) of 4–8-cell stage and extended to the blastocyst stage, we performed high-throughput sequencing on *in vitro*-matured (16.5 h) MII oocytes. We found that the pluripotent gene expression of ID1 and SOX2 was blocked in the low-calcium model, which may be related to the poor development ability of SCNT embryos.

## Materials and Methods

All chemicals and reagents for this study were purchased from Sigma–Aldrich (St. Louis, MO, United States), unless otherwise indicated. Protocols for all animal studies were approved by the Institutional Animal Care and Use Committee of China Agricultural University.

### Oocyte Culture and Sample Collection

Bovine ovaries were collected at a local slaughterhouse. Cumulus–oocyte complexes (COCs) were collected from antral follicles (3–8 mm in diameter). COCs were selected using a microscope (SZ61, Olympus, Tokyo, Japan) and washed three times in maturation medium containing tissue culture medium 199 (TCM199) (GIBCO-BRL, Grand Island, NY, United States), supplemented with 10% (*v*/*v*) fetal bovine serum (FBS) (GIBCO-BRL, Grand Island, NY, United States), 0.02 IU/ml follicle-stimulating hormone (FSH) (Sioux Biochemical), 0.02 IU/ml luteinizing hormone (LH) (Sioux Biochemical), and 1 mg/ml β-estradiol. After washing, the COCs were cultured in maturation medium with or without 50 μM BAPTA-AM (in the form of 50-mM stocks in DMSO) for 24 h at 38.5°C at 5% CO_2_ in humidified air. Then, COCs were removed by gently pipetting in Dulbecco’s phosphate-buffered saline (DPBS) (GIBCO-BRL, Grand Island, NY, United States) containing 0.1% (*w*/*v*) hyaluronidase and then used for the next series of experiments.

### Somatic Cell Nuclear Transfer and Culture

After IVM for 16.5 h, denuded oocytes (DOs) were obtained from COCs by gently pipetting in DPBS containing 0.1% (*w*/*v*) hyaluronidase, and the first polar body was selected using a microscope (SZ61, Olympus, Tokyo, Japan) at × 20 magnification. MII oocytes were transferred into a droplet of buffer solution containing TCM199, supplemented with 10% (*v*/*v*) FBS, 7.5 mg/ml of cytochalasin B (Calbiochem 250233). Oocytes undergoing micromanipulation were held with a holding pipette, the zona pellucida was cut with a glass needle, and the MII chromosome–spindle complex was pushed out. After enucleation, oocytes were washed three times with a buffer solution and returned to the incubator. The donor nuclei were gently aspirated in and out of the injection pipette until the nuclei were largely devoid of visible cytoplasmic material. Each nucleus was injected into a separate enucleated oocyte. Following somatic cell nucleus injection, oocytes were activated by culturing in Ca^2+^-free CZB containing 10 mM Sr^2+^ and 5 μg/ml cytochalasin B for 5 h and cultured at 37°C under 5% (vol/vol) CO_2_ in air.

### Ca^2+^ Imaging

Oocytes from different treatment groups at 0, 8, 12, and 24 h during IVM were washed three times with a washing medium [DPBS with 3 mg/ml of bovine serum albumin (BSA)]; after which, they were incubated in a washing medium with 5 μM Fluo-3/AM (Invitrogen/Molecular Probes, Carlsbad, CA, United States) for 30–40 min in the dark at 39°C in 5% CO_2_ and humidified air and washed three–five times to remove the Fluo-3/AM. Oocytes were allocated to 6-μl drops of DPBS under mineral oil and were ultimately observed using a Live Cell Imaging System (Nikon A1, Nikon, Tokyo, Japan). The [Ca^2+^]_i_ of oocytes was detected 10 times at intervals of 20 s using the fluorescence intensity of the laser at 488 nm (F488), and results were analyzed with software from NIS-Elements (Nikon, Tokyo, Japan).

### Immunofluorescence and Live Cell Imaging

Metaphase II oocytes were incubated in DPBS containing 2.5% pronase for 2–3 min in order to remove their zona pellucida (ZP). Then, ZP-free oocytes were incubated with ER-Tracker Red (1:500) (Beyotime Institute of Biotechnology, China) or Mito-Tracker Green (1:1,000) (Beyotime Institute of Biotechnology, China) at 39°C, 5% CO_2_ for 30 min. Oocytes were washed three times and imaged using a Live Cell Imaging System (Nikon A1, Nikon, Tokyo, Japan). For live imaging, oocytes were isolated under mineral oil and imaged using A1, scanning the *Z*-axis of oocytes with the same depth (10 μm) and seven steps, and the maximum intensity projections of the equatorial cross-section of the oocytes were showed.

### Quantitative Reverse Transcription PCR

Quantitative reverse transcription PCR (RT-qPCR) was performed by using an Applied Biosystems Step One Plus System and Power SYBR Green PCR Master Mix (TransGen Biotech). RNA was extracted from 50 to 70 oocytes using QIAGEN RNeasy Mini Kit, and cDNA was made by using High Capacity cDNA Reverse Transcription Kit (Applied Biosystems). cDNA was treated with RNase H and diluted 1:10 in H_2_O, with 8 μl used per PCR. Gapdh was used as a control. Real-time PCR was performed as SYBR Green assays using an ABI 7500 real-time PCR instrument (Applied Biosystems). Primers used in this assay are designed using the software Primer Premier v5.0 (Premier Biosoft International) and shown in Supplementary Table 1. Experiments were performed in biological triplicate and technical duplicate, with data represented as means ± SEM.

### ATP Content Assay

The level of ATP in each oocyte was measured by using an Enhanced ATP Assay Kit, S0027 (Beyotime Institute of Biotechnology, China). Firstly, different degrees of ATP standard were prepared, ranging from 0 to 40 pmol ATP. According to the manufacturer’s instructions, oocytes were treated with 20 μM lysis buffer, and then, lysed cells were centrifuged for 5 min at 4°C and 12,000*g*. All processes were handled on ice. An ATP-detecting solution was added to 96-well plates, and response at room temperature for 3–5 min was observed. Secondly, standard solutions and ATP detection diluent were added into each well. Then, samples were also added into each well, and luminescence signals were immediately calculated using a luminometer (Infinite F200; Tecan). Then, the ATP content of every oocyte can be calculated from the standard curve (pmol/oocyte).

### Reactive Oxygen Species Assay

To analyze the levels of intracellular ROS in bovine oocytes, a Reactive Oxygen Species Assay Kit was applied to detect ROS as a green fluorescent DCFH-DA signal. Oocytes of each group were incubated in 10 μmol/l DCFH-DA (in a maturation medium) for 30 min at 38.5°C. After washing three times in D-PBS containing 0.1% BSA, the samples were placed on glass dishes, and the fluorescence intensity of each oocyte was measured under a fluorescence microscope (Olympus IX73, Tokyo, Japan) and quantified using EZ-C1 Free-Viewer (Nikon).

### Annexin-V Analysis

To detect the externalization of phosphatidylserine in early apoptotic oocytes, Annexin V-FITC staining was performed using an Annexin V-FITC/EGFP Apoptosis Detection Kit (Vazyme Biotech Co., Ltd., Nanjing, China) according to the manufacturer’s instructions. Briefly, the oocytes from each group were incubated in 100 μl binding buffer containing 10 μl of Annexin V-FITC for 30 min in the dark after washing twice in TCM-199. The oocytes were transferred to a TCM-199 drop in living cell culture dishes. The Annexin-V fluorescent signals were measured with a fluorescence microscope (Olympus IX73, Tokyo, Japan).

### JC-1

To monitor mitochondrial membrane potential (Δφm), the Mitochondrial Membrane Potential Assay Kit (Beyotime Institute of Biotechnology, China) came into use. Oocytes were incubated to 10 μM JC-1 in 100 μM working solution at 37.0°C in 5% CO_2_ for 20 min. Then, the oocytes were washed with washing buffer to remove surface fluorescence and observed using a fluorescence microscope (Olympus IX73, Tokyo, Japan). The J-aggregate (red)/monomer (green) fluorescence ratio was calculated to represent Δφm.

### RNA-Seq

Metaphase II oocytes were incubated in DPBS containing 2.5% pronase for 2–3 min in order to remove their ZP. Then, ZP-free oocytes were carefully washed several times with DPBS, transferred to cell lysis buffer containing RNase inhibitor, and stored at −80°C. The MII oocytes were directly lysed and used for cDNA amplification by Smart-Seq2. RNA-seq was carried out by MicroAnaly Gene Technologies Co., Ltd., (Shanghai, China). Briefly, after amplification, the cDNA samples were purified using Beckman AMPure XP, and their concentration was identified by Qubit^®^ 3.0 Fluorometer (Life Technologies, CA, United States). Then, the distribution of fragments and quality of amplified products were determined using an Agilent 2100 High Sensitivity DNA Assay Kit (Agilent Technologies, CA, United States). To construct the sequencing libraries, 20 ng of amplified cDNA from each sample was broken to a small fragment about 300 bp using Bioruptor^®^ Sonication System (Diagenode Inc.), the fragmented cDNAs were end-repaired, and a single A base was added to the 3′-end and ligated with a sequencing adapter. Then, after each reaction, purification was performed using Beckman AMPure XP, adding different index tags to each sample during the sequencing process to distinguish each other. These cDNAs were then amplified by PCR, and the PCR amplification products were electrophoresed on 2% agarose gels. DNA fragments of ≥4,000 bp were recovered by CWBIO Gel Extraction Kit and dissolved in EB buffer to obtain the final sequencing libraries. The sequencing libraries were assessed using the Agilent 2100 Bioanalyzer (Agilent Technologies), and the Bio-Rad Kit iQ SYBR Green of Bio-Rad CFX 96 Real-Time PCR system was used to accurately quantify the qualified insert size (library valid concentration > 2 nm) after the insert size was in line with expectations. The qualified cDNA libraries were sequenced on the Illumina HiSeq X Ten platform (Illumina, San Diego, CA, United States) with read lengths of paired-end 150 bp. Sequencing reads were filtered by removing the reads containing adapters or having more than 1% unrecognizable bases and low-quality reads before mapping. Clean reads were mapped to the bovine genome Bos_taurus_UMD_3.1.90 (Ensembl). Data were normalized to reads per kilobase per million reads (RPKM) by transforming uniquely mapped transcript reads. Three biological replicates were performed for each group, and a total of six samples were sequenced. Genes that were differently expressed between the control and treated oocyte groups were determined using DEseq2 with two cut-offs: an adjusted *P*-value of 0.05 and a minimum fold change of 2.

### Statistical Analysis

All experiments were repeated at least three times. Data are presented as means ± SEM, unless otherwise stated. All data for the different groups were analyzed using SPSS 17.0 software (SPSS, Inc., Chicago, IL, United States) and Student’s *t*-test. *P* < 0.05 was considered to be statistically significant.

## Results

### The Buildup of the Lower Calcium Model

**FIGURE 1:**
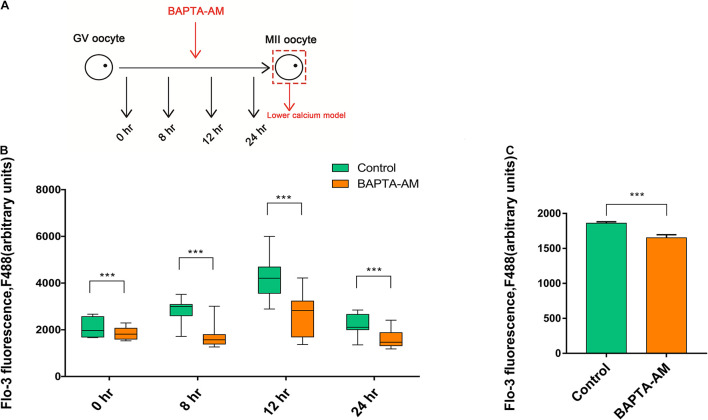
The buildup of the lower calcium model. **(A)** Schematic illustration of the experimental approach. **(B)** The level of [Ca^2+^]_i_ in oocytes at 0, 8, 12, and 24 h during *in vitro* maturation (IVM) treated with or without BAPTA-AM (50 μM). **(C)** The [Ca^2+^]_i_ level of metaphase II (MII) oocytes contained the first polar body at 16.5 h of IVM. All experiments were performed in at least triplicates, and the data represent the means ± SEMs, ***P < 0.001.

A low-calcium model of bovine MII-stage COCs was established by adding 50 μM BAPTA-AM into *in vitro* maturation culture system (Figure 1A). To test the efficiency of the low-calcium model, a specific calcium ion fluorescent probe Fluo-3-AM was used to indicate the intracellular [Ca^2+^]_i_ level of bovine oocytes. Results showed that the [Ca^2+^]_i_ level was significantly decreased at 0, 8, 12, and 24 h after BAPTA-AM treatment when compared with the control group (0 h: control group 2,075.27 ± 46.09, oocyte number *n* = 70 vs. treatment group 1,836.79 ± 27.78, *n* = 80, *P* < 0.001; 8 h: control group 2,804.34 ± 69.74, *n* = 60 vs. treatment group 1,760.33 ± 72.56, *n* = 60, *P* < 0.001; 12 h: control group 4,243.22 ± 96.26, *n* = 80 vs. treatment group 2,685.96 ± 97.75, *n* = 80, *P* < 0.001; 24 h: control group 2,200.49 ± 52.63, *n* = 70 vs. treatment group 1,608.42 ± 41.56, *n* = 80, *P* < 0.001; Figure 1B).

Somatic cell nuclear transfer was conducted when MII oocytes matured *in vitro* for 16.5 h and expelled the first polar body. Hence, 16.5 h was selected to explore the intracellular [Ca^2+^]_i_ level. In Figure 1C, the intracellular [Ca^2+^]_i_ level of 16.5 h was significantly lower in the BAPTA-AM treatment group when compared with the control group (control group 1,861 ± 20.49, *n* = 50 vs. treatment group 1,652 ± 43.9, *n* = 40, *P* < 0.001; Figure 1C). These results indicated that intracellular calcium ion chelating agent BAPTA-AM can successfully establish an MII low-calcium model of bovine oocytes.

### Effect of Low Calcium on Nuclear Maturation and Somatic Cell Nuclear Transfer Embryos

**FIGURE 2:**
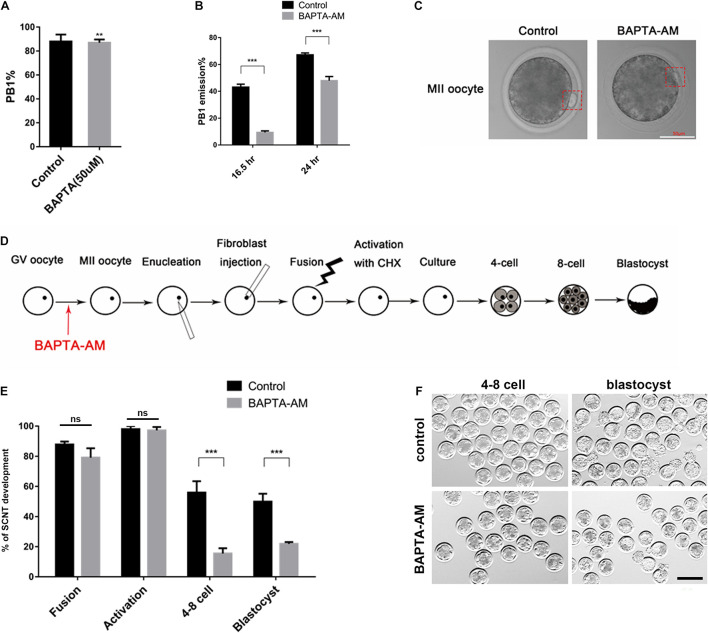
The effect of continuous low calcium on nuclear maturation and SCNT embryos. **(A)** The rate of PB1 extrusion at the 16.5 h of IVM. **(B)** The rate of PB1 at 16.5 and 24 h of IVM. **(C)** The morphology of MII oocytes, and the PB1 is marked by red dashed rectangle (scale bar, 50 μm). **(D)** Schematic illustration of the experimental approach. **(E)** The rate of SCNT embryo development (% of fusion = the number of fusion/the number of enucleation, % of activation = the number of activation/the number of fusion, % of 4–8 cell = the number of embryos at 4–8-cell stage/the number of activation, % of blastocyst = the number of blastocyst/the number of embryos at 4–8-cell stage). **(F)** Representative images of SCNT embryos after 48 and 168 h of culturing *in vitro*; normal embryos are shown in the red dotted line (scale bar, 200 μm). All experiments were performed in at least triplicates, and the data represent the means ± SEMs. **P < 0.01, ***P < 0.001; ns, no significant difference.

After 16.5 h of IVM culture, the proportion of oocytes with PB1 was significantly reduced in the low-calcium model group (control group 92.32 ± 0.94, *n* = 1,483 vs. treatment group 87.14 ± 1.45, *n* = 969, *P* < 0.01; Figure 2A). Meanwhile, we found that part of the PB1 in the low-calcium model group was not completely expelled when compared with the control group (Figure 2B). At 16.5 and 24 h, the proportion of completely expelled PB1 oocytes was calculated, respectively. Results showed that the proportion of completely expelled PB1 oocytes in the low-calcium group was significantly lower than that in the control group (16.5 h: control group 43.80 ± 1.38, *n* = 979 vs. treatment group 10.90 ± 0.76, *n* = 879, *P* < 0.001; 24 h: control group 67.28 ± 1.35, *n* = 716 vs. treatment group 48.53 ± 1.36, *n* = 712, *P* < 0.001; Figure 2B).

To investigate the reprogramming ability in low-calcium model, we performed SCNT and then obtained clone embryos. Results showed that there was no difference in the fusion rate and activation rate between the low-calcium model group and the control group during the SCNT process, but the 4:8 cell ratio and blastocyst rate were significantly reduced (fusion ratio: control group 87.85 ± 1.92, *n* = 510 vs. treatment group 79.13 ± 6.19, *n* = 528, ns; activation ratio: control group 98.22 ± 1.54, *n* = 470 vs. treatment group 97.76 ± 1.79, *n* = 416, ns; 4:8 cell ratio: control group 56.96 ± 6.87, *n* = 462 vs. treatment group 15.47 ± 3.51, *n* = 410, *P* < 0.01; blastocyst rate: control group 51.68 ± 4.56, *n* = 307 vs. treatment group 18.46 ± 2.12, *n* = 87, *P* < 0.01; Figure 2E). There was also a large number of abnormal embryos with fragmentation or uneven cleavage at the 4–8-cell stage in the low-calcium model (Figure 2E). Compared with the control group, blastocysts formed in low-calcium model were less likely to hatch normally (Figure 2F). The data in Figure 2 therefore suggest that low calcium blocked polar body complete extrusion and exhibit a low developmental competence. Although the low-calcium model clones showed no abnormalities during the fusion and activation stage, when entering the ZGA such as the 4–8-cell stage, the embryo development rate was significantly lower than that of the control group, and the effect continued to the blastocyst stage.

### Cytoplasmic Maturation of Oocytes Was Impaired Under Low-Calcium Model

**FIGURE 3:**
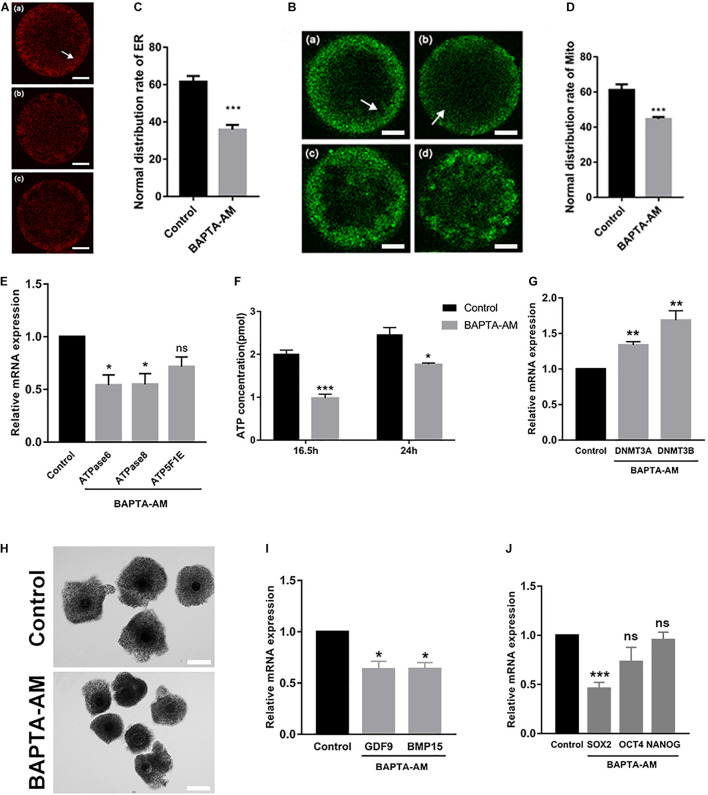
Cytoplasmic maturation of oocytes was impaired under low-calcium model. **(A–D)** Representative images and the ratios of different ER and Mito distribution patterns in oocytes. The ER and Mito profile is on the left. Normal distribution of ER **(a)** and Mito **(a)**: they were uniformly distributed in clusters in the cortex (white arrow); **(b)**, **(c)** and **(c)**, **(d)** are abnormal distribution of ER and Mito: they were distributed in non-uniform clusters in the cortex. The proportion of ER and Mito normal distribution between the control group and the low-calcium group is shown on the right (scale bar, 20 μm). **(E)** ATPase6, ATPase8, and ATP5F1E mRNA levels in oocytes matured for 16.5 h *in vitro*. **(F)** ATP content in oocytes matured for 16.5 and 24 h *in vitro*. **(G)** mRNA expression levels of DNA methyltransferases DNMT3A and DNMT3B in oocytes matured for 16.5 h *in vitro*. **(H)** The oocyte cumulus extension matured for 16.5 h *in vitro* (scale bar, 100 μm). **(I)** Oocyte maturation-related gene and **(J)** pluripotency gene mRNA expression levels in oocytes matured for 16.5 h *in vitro*. All experiments were performed in at least triplicates, and the data represent the means ± SEMs. *P < 0.05, **P < 0.01, and ***P < 0.001; ns, no significant difference.

Cytoplasmic maturation of oocytes has a great influence on the subsequent development of embryos. Fluorescence localization of two important calcium-related organelles, the endoplasmic reticulum and the mitochondria, showed that they were distributed in non-uniform clusters in cortical regions in low-calcium MII oocytes, and the abnormal proportion was significantly decreased (ER: control group 61.57 ± 3.05, *n* = 42 vs. treatment group 35.89 ± 2.51, *n* = 46, *P* = 0.001; Figures 3A,B; Mito: control group 61.173 ± 3.21, *n* = 39 vs. treatment group 44.66 ± 1.11, *n* = 38, *P* < 0.01; Figures 3C,D). Moreover, mitochondrial-related genes ATPase6 and ATPase8 were downregulated in low-calcium oocytes (*P* < 0.05; Figure 3E), while ATP5F1E was not changed. At the same time, ATP content was significantly decreased in the low-calcium group, indicating that mitochondrial function was impaired (16.5 h: control group 2.02 ± 0.08 pmol, *n* = 24 vs. treatment group 0.98 ± 0.09 pmol, *n* = 24, *P* < 0.001; 24 h: control group 2.47 ± 0.16 pmol, *n* = 24 vs. treatment group 1.76 ± 0.03 pmol, *n* = 24, *P* < 0.05; Figure 3F).

After 16.5 h of IVM, cumulus expansion of COCs in the low-calcium group was significantly insufficient, as shown in Figure 3H. The expression of genes related to cytoplasmic maturation in low-calcium MII oocytes was significantly lower than that in the control group (*P* < 0.05; Figure 3I), and the pluripotent factor SOX2 was also significantly decreased under low calcium (*P* < 0.001; Figure 3J). DNMT3a and DNMT3b are demethylases, and their mRNA expression levels were significantly increased in the low-calcium group (*P* < 0.01; Figure 3G), which may be the reason why some key genes have failed to activate. In general, the abnormal COC morphology, impaired distribution of endoplasmic reticulum and mitochondria, as well as the expression and activation of key genes were all changed in low-calcium oocytes, indicating that cytoplasmic maturation is severely impaired under low-calcium condition.

### Low Calcium Can Induce Oxidative Stress in Oocytes and Lead to Early Apoptosis

**FIGURE 4:**
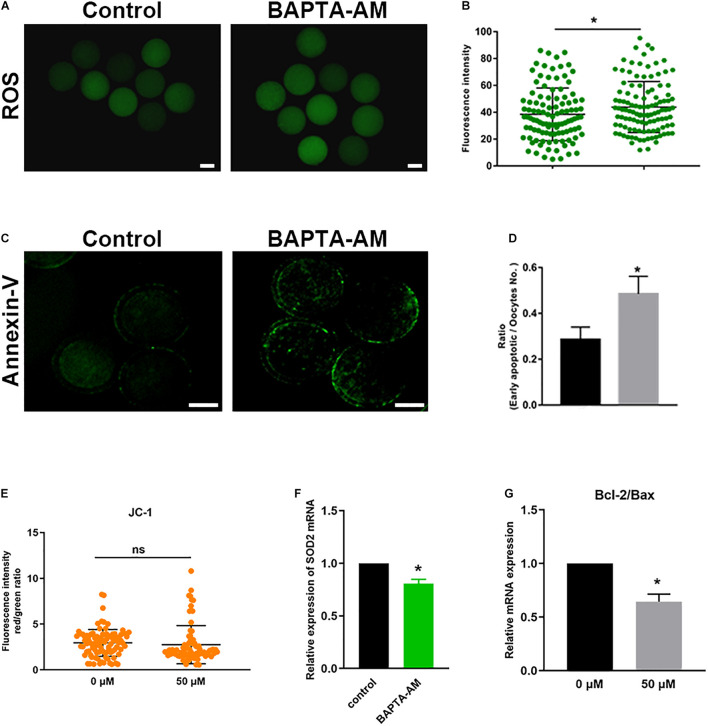
Low calcium can induce oxidative stress in oocytes and lead to early apoptosis. **(A)** The reactive oxygen species (ROS) level in the control and low calcium groups of oocytes matured for 16.5 h *in vitro* (scale bar, 100 μm). **(B)** ROS fluorescence intensity analysis. **(C)** SOD2 mRNA expression levels in oocytes matured for 16.5 h *in vitro*. **(D)** Annexin-V signals in the control and low-calcium groups of oocytes matured for 16.5 h *in vitro* (scale bar, 50 μm). **(E)** The proportion of early apoptotic oocytes. **(F)** Bcl-2/Bax mRNA expression levels in oocytes matured for 16.5 h *in vitro*. **(G)** JC-1 fluorescence intensity analysis. All experiments were performed in at least triplicates, and the data represent the means ± SEMs. *P < 0.05; ns, no significant difference.

To detect the ROS level, DCHF-DA fluorescent dye was used in each group. As shown in Figures 4A,B, the oxidative stress in the low-calcium group was significantly increased (control group 38.47 ± 1.89, *n* = 107 vs. treatment group 43.86 ± 1.78, *n* = 113; *P* < 0.05). SOD2 is located in the mitochondria, which is the most important antioxidant enzyme to remove intracellular ROS. The mRNA expression of SOD2 was significantly reduced (*P* < 0.05; Figure 4F), indicating that the antioxidant capacity of oocytes was impaired. Oocyte oxidative stress may lead to apoptosis, so Annexin-V was used to detect the early apoptosis of oocytes. Results showed that the low-calcium group exhibits a higher ratio of early apoptosis (control group 0.29 ± 0.05, *n* = 68 vs. treatment group 0.49 ± 0.07, *n* = 70; *P* < 0.05; Figures 4C,D). The next step, after detection of the JC-1, found no significant differences in the two groups (control group 2.95 ± 0.15, *n* = 94 vs. treatment group 2.87 ± 0.26, *n* = 78; *P* < 0.05; Figure 4E). Besides, the Bcl-2/Bax mRNA ratio in the low-calcium group decreased significantly (*P* < 0.05; Figure 4G), indicating that low calcium status can lead to oxidative stress and early apoptosis in bovine oocytes.

### RNA-Seq of Bovine *in vitro* Maturation 16.5-h Oocytes Under Low-Calcium Model

**FIGURE 5:**
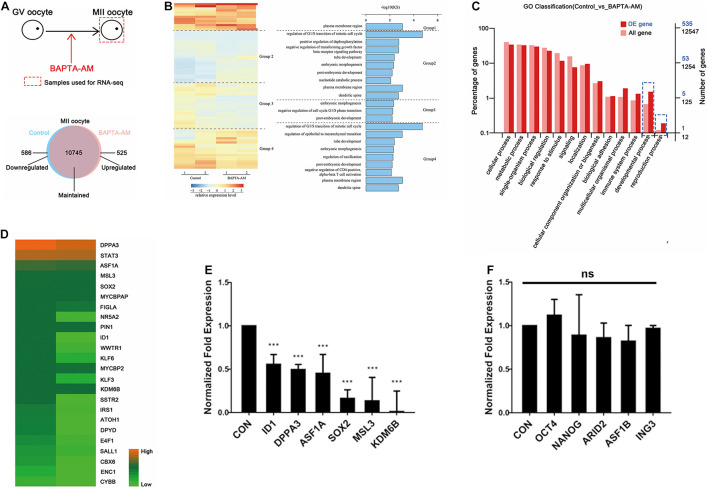
RNA-seq of bovine IVM 16.5-h oocytes under low-calcium model. **(A)** Schematic diagram of experimental method and Venn diagram of differentially expressed genes (DEGs). **(B)** Clustering heat map of DEGs between the control group and the low-calcium group. Seventy-nine DEGs (*P* value < 0.05, FC > 2) were divided into four groups by unsupervised clustering. The right side is corresponding to the GO analysis effect diagram of the four groups of DEGs. KS < 0.01. **(C)** Gene ontology analysis of DEGs and all genes. **(D)** Twenty-four pluripotency-related differential genes were screened in the downregulated gene region. **(E)** The mRNA expression level of pluripotent genes was significantly downregulated. **(F)** The mRNA expression level of pluripotent genes was not significantly changed. Data are means ± SEM. ***P < 0.001; ns, no significant difference.

To further characterize the mechanism of low-calcium damage to bovine oocytes, RNA-seq was performed on MII oocytes at 16.5 h of IVM. The differentially expressed genes between the low-calcium model and the control group were screened: among the detected 10,555 genes (FPKM > 0.1), there were 79 differentially expressed genes (DEGs) (*P* value < 0.05, FC > 2) between the low-calcium model and the control group (Figure 5A). Among them, compared with the control group, 51 downregulated genes and 28 upregulated genes were found in the low-calcium model (Figure 5A). Gene ontology (GO) analysis was performed on the selected DEGs, and it was found that the significant enrichment of DEGs was related to the regulation of G1/S transition during the cell cycle of mitosis (KS < 0.01) (Figure 5B). The gene with the most significant difference in this enriched item was ID1. However, a GO analysis was performed on the selected DEGs and all detected genes; the blue dotted box shows the developmental process and function of the reproductive process proportion obvious differences, namely, DEGs and all genes’ enrichment trend is different. It can be inferred that DEGs play an important role, which is consistent with our conclusion that the low-calcium model has a lower developmental ability of clonal embryos (Figure 5C). The upregulated and downregulated genes were analyzed by KEGG. The pluripotency-related pathway enrichment genes were mainly located in the downregulated gene region. Then, 24 pluripotency-related differential genes were screened in the downregulated gene region (Figure 5D). Six genes that have been identified as important pluripotency genes were quantitatively verified, and the results were consistent with transcriptome sequencing results (Figure 5E). At the same time, five important pluripotency-related genes that showed no change in transcriptome sequencing were further verified by qPCR (Figure 5F). Results showed that only a fraction of pluripotency genes was expressed in the oocyte by calcium regulation, such as recognized pluripotency genes OCT4, NANOG, and SOX2. Among the three, only SOX2 expression is regulated by calcium ions, and these regulated pluripotency genes, in turn, influence the reprogramming ability of oocytes. Our study indicates that intracellular calcium is an important maternal factor for oocyte reprogramming.

## Discussion

In this study, we explored the effects of low calcium levels on maturation and reprogramming in bovine oocytes. We found that the decrease of cytoplasmic calcium leads to arrested nuclear maturation of oocytes, impaired cytoplasmic maturation, and reduced developmental ability of SCNT embryos. Low cytoplasmic calcium impairs mitochondrial function, as well as oxidative stress and apoptosis. These results indicate that calcium ion is an important factor affecting the maturation and reprogramming of bovine oocytes.

Somatic cell nuclear transfer serves as an important assisted reproductive technology, but the overall efficiency is low so far. Studies have shown that oocyte quality is directly related to the reprogramming ability of animals, and it is an important factor affecting the efficiency of animal cloning and *in vitro* fertilization. Changes of calcium ions in oocytes play an important regulatory role in the process of oocyte maturation. In this study, we used the intracellular calcium ion specific chelator BAPTA-AM to investigate the changes of the developmental potential and reprogramming ability in oocytes from the perspective of [Ca^2+^]_i_ level. [Ca^2+^]_i_ level was reduced by BAPTA-AM (50 μM) to delay the age-induced developmental decline of bovine oocytes (Zhao et al., 2015). In our previous studies, 50 μM BAPTA-AM was added into a culture medium in which grade-3 COCs are incubated, which not only improved the quality of oocytes but also improved the developmental ability of *in vitro* fertilization and parthenogenetic-activated embryos (Hu et al., 2018). Therefore, the same concentration of BAPTA-AM was also used in this study to construct a low-calcium model in bovine oocytes. Results also elucidated that the [Ca^2+^]_i_ level of oocytes was significantly decreased at 0, 8, 12, and 16.5 h of *in vitro* maturation after BAPTA-AM treatment, which achieved the purpose of continuously reducing the [Ca^2+^]_i_ level in oocytes (Figure 1).

From our research, PB1 extrusion and SCNT embryo development in the low-calcium model were inhibited. BAPTA-AM was found to affect the development of GVBD in porcine and bovine oocytes and could prevent or delay FSH-induced oocyte maturation (Homa et al., 1991; Grazul-Bilska et al., 1997). These results illustrated that Ca^2+^ plays an important role in oocyte maturation. The fusion rate and activation rate of the low-calcium model were not affected during SCNT (Figure 2E). As we all know, the ZGA period of cattle is the 4–8-cell stage, and we found that the cloned embryo developmental rate of the low-calcium model was decreased significantly from the ZGA stage to the blastocyst stage, which was obtained from SCNT (Figure 2E). These results suggest that [Ca^2+^]_i_ is one of the factors affecting the reprogramming ability of oocytes.

We have also carried out related studies of oocyte cytoplasmic maturation, which is an important indicator for oocyte maturation. The endoplasmic reticulum and the mitochondria are the store of calcium ions. Research revealed that endoplasmic reticulum clustered in the cortex when oocytes matured, which prepared for subsequent fertilization [Ca^2+^]_i_ oscillation (Stricker, 2006). The migration of the ER to the cortex during maturation is thought to play an important role in rendering the ER competent to generate the calcium transients, and the redistribution of ER is believed to be primarily mediated by microtubules and microfilaments (Kim et al., 2014). When bovine oocytes were treated with calcium ionophore (A23187), the spindle was disassembled, and microtubule networks were distributed in the oocyte cytoplasm (Liu et al., 1998). These suggest that the abnormal distribution of ER may be the reason for microtubule abnormalities caused by low calcium (Figures 3A,B). The mitochondria regulate calcium uptake in response to cytoplasmic calcium signals (Fan et al., 2020). In the present study, the proportion of the mitochondria with normal distribution was decreased in the low-calcium model (Figures 3C,D). This abnormal distribution pattern may be a response to a decrease in cytoplasmic calcium. The mitochondria produce ATP through oxidative phosphorylation, which provides energy for events such as fertilization of oocytes. The key enzyme for ATP production is ATP synthase, which has two subunits. F0 is encoded by ATP synthase F0 subunit 6 (Atpase6) and ATPase F0 subunit 8 (Atpase8), while ATP5F1E is involved in encoding F1 subunit (Ridlo et al., 2021). Therefore, the synthesis of ATP can be increased by increasing the expression of Atpase6 and Atpase8. In addition to abnormal distribution, the ATP content of oocytes under low calcium was extremely significantly reduced compared to the control group (Figure 3D). The mRNA levels of genes encoding ATP-related enzymes (Atpase6, Atpase8, and ATP5F1E) were also reduced (Figure 3C), indicating impaired mitochondrial function.

Studies have shown that during *in vitro* maturation of COCs, various metabolic and hormonal factors expressed by cumulus cells are positively correlated with *in vitro* maturation of oocytes (Turhan et al., 2021). Cumulus cells are very important for achieving oocyte development (Zhang et al., 1995; Tanghe et al., 2003; Ortiz-Escribano et al., 2016), which can mediate the nuclear and cytoplasmic maturation of oocytes, maintain oocytes in the state of meiotic arrest, and participate in the induction of meiotic recovery (Fukui and Sakuma, 1980; Chian et al., 1994; Tanghe et al., 2002). Therefore, cumulus expansion is often used as a factor to indicate oocyte quality. Our results revealed that the cumulus cell expansion of bovine oocytes under low calcium was significantly weaker than that of the control group, which probably affected the meiotic resumption and cytoplasmic maturation of oocytes through their gap junctional network (Figure 3F). Oocytes actively participate in follicular development, including proliferation and differentiation of granulosa cells *via* oocyte-derived factors: GDF9 and BMP15, which, with surrounding granulosa cells, probably form a communication loop using these molecules (Alam and Miyano, 2020). The mRNA levels of GDF9 and BMP15 in low calcium, which ensured the coordinated oocyte growth, were decreased (Figure 3G). At the same time, the mRNA level of SOX2 gene in the pluripotent factor was also extremely significantly decreased (Figure 3H), which could be responsible for defective SCNT embryonic development. Our results indicated the low calcium resulted in severe damage to oocyte cytoplasm.

Next, we discussed the possible reasons for the reduced maturation and reprogramming ability of bovine oocytes after calcium ion reduction. Mitochondrial dysfunction can induce excessive ROS production, resulting in oxidative stress and damage to cells (Li et al., 2020). Therefore, we detected ROS levels in the low-calcium model, and the results showed that low calcium led to the excessive production of ROS (Figures 4A,B), and SOD2, as the most important antioxidant enzyme to remove ROS in cells, mRNA expression level was significantly reduced (Figure 4C), and oxidative stress was induced. However, the production of a large number of ROS can trigger cell apoptosis (Li et al., 2010), and our statistical results also showed that the proportion of early apoptosis of oocytes was significantly increased under low calcium (Figure 4D). Bax triggers opening of the mitochondrial permeability transition pore (PTP) has been proposed as one of the models of mitochondrial matrix swelling and rupture of the outer membrane (Antonsson, 2001). PTP is involved in Bax-induced apoptosis accompanied by reduced mitochondrial membrane potential (MMP) (Vander Heiden et al., 1997; Pottecher et al., 2013). As shown in Figure 4E, the BAPTA-AM treatment group exhibited decreased MMP, but there was no statistical difference (control group 2.95 ± 0.15, *n* = 94 vs. treatment group 2.87 ± 0.26, *n* = 78; *P* < 0.05). Meanwhile, immunofluorescent staining and RT-qPCR results showed that BAPTA-AM treatment induces early apoptosis proved by Annexin-V and Bcl2/Bax mRNA level, respectively. We hypothesize that the decrease in MMP caused by BAPTA-AM treatment is accompanied by the release of pro-apoptotic factors into the cytoplasm, which, together with the death receptor pathway, mediate oocyte apoptosis. So, we speculate that calcium ion reduction can induce ROS and early apoptosis.

Further RNA-seq sequencing was performed on the low-calcium model and control groups, and 79 DEGs were screened out, including 51 downregulated genes and 28 upregulated genes (Figure 5A). Through GO functional classification analysis, it was found that the significantly enriched DEGs were related to the regulation of G1/S conversion in the process of mitotic cell cycle (Figure 5B). The gene with the most significant difference in this enriched item was ID1, which was further screened out and verified by real-time quantitative PCR (Figure 5E). At present, the role of ID1 in G1/S transformation of cell cycle has been confirmed, and studies have found that ID1 accelerates the process of cell cycle transition from G1 to S phase by promoting the expression of Wnt2 (Ling et al., 2014; Xia et al., 2016). Then, 24 pluripotency-related differential genes were screened in the downregulated gene region (Figure 5D). Six genes that have been identified as important pluripotency genes were quantitatively verified. It is known that the reactivation of pluripotent genes is an important event for the successful reprogramming of somatic cell nuclei to a pluripotent state. OCT4, NANOG, and SOX2 are three important pluripotent genes (Gurdon and Wilmut, 2011). In this study, it was found that the mRNA level of SOX2, an important downstream pluripotency gene, was significantly reduced, but OCT4 and NANOG did not change (Figures 5E,F). This result is consistent with the results of a 2013 study, which found that SOX2 was significantly downregulated after ID1 interference in glioma cell lines (Soroceanu et al., 2013). Therefore, it can be speculated that the decrease of SOX2 in the low-calcium model may be related to ID1. Based on the above results, it is speculated that [Ca^2+^]_i_ probably affects the reprogramming ability of bovine oocytes through ID1.

## Conclusion

In conclusion, our results reported that calcium ions had an important effect on the maturation and reprogramming of bovine oocytes. In low-calcium group oocytes, BAPTA-AM induced impaired cytoplasmic maturation and nuclear maturation by inducing oxidative stress and early apoptosis, thus reducing the maturation and reprogramming ability of bovine oocytes. These negative effects may be caused by the reduction expression of ID1.

## Data Availability Statement

The data presented in the study are deposited in the NCBI BioProject repository, accession number PRJNA761245 (BioSample accessions: SAMN21246665, SAMN21246666, SAMN21246667, SAMN21246668, SAMN21246669, and SAMN21246670).

## Ethics Statement

The animal study was reviewed and approved by the Institutional Animal Care and Use Committee of China Agricultural University.

## Author Contributions

LM and HH designed the experiments and participated in animal experiments. ZL participated in animal research. LZ, QZ, and XL prepared the figures and partial experiment. XF contributed to the writing and revising of the manuscript. All authors contributed to the article and approved the submitted version.

## Conflict of Interest

The authors declare that the research was conducted in the absence of any commercial or financial relationships that could be construed as a potential conflict of interest.

## Publisher’s Note

All claims expressed in this article are solely those of the authors and do not necessarily represent those of their affiliated organizations, or those of the publisher, the editors and the reviewers. Any product that may be evaluated in this article, or claim that may be made by its manufacturer, is not guaranteed or endorsed by the publisher.
