# Supplementary material for: The Role of Ca2 + in Maturation and Reprogramming of Bovine Oocytes: A System Study of Low-Calcium Model
Source: Front Cell Dev Biol. 2021 Oct 26;9:746237. doi: 10.3389/fcell.2021.746237 (PMC8577575; doi:10.3389/fcell.2021.746237)
Supplement: Supplementary file 1 [file Data_Sheet_1.PDF]

Supplementary Table 1 Oligonucleotide primer sequences used for quantitative Real-Time PCR

Genes	Accession	number Primer sequences(5'-3')	
GAPDH	NM_001034034.2	F	CTGCCACCCAGAAGACTGTGGATGG
		R	ACAGACACGTTGGGAGTGGGGACG
ID1	NM_001097568.2	F	GCTGCTACTCGCGCCTC
		R	GATTTCGCCGTTGAGAGTGC
SOD2	NM_201527.2	F	GGGAGAATGTAACTGCACGA
		R	ACAACAGAGCAGCGTACTGG
ATPase6	YP_209210.1	F	GAACACCCACTCCACTAATCCCAAT
		R	GTGCAAGTGTAGCTCCTCCGATT
ATPase8	YP_209209.1	F	CACAATCCAGAACTGACACCAACAA
		R	CGATAAGGGTTACGAGAGGGAGAC
ATP5F1E	NM_001143741.1	F	CAGGCTGGACTCAGCTACATC
		R	AGTCTTCATGGCGTTTGCTT
DNMT3A	NM_001206502.2	F	GGA CAA GAA TGC CAC CAA AG
		R	CTC GTT GGG TCA TGT GGT T
DNMT3B	NM_181813.2	F	GGG AAG GAG TTT GGA ATA GGA G
		R	GGT GAT GGC AAG TTC TCC G
GDF9	NM_174681.2	F	CCATGGCGCTTCCCAACAAAT
		R	CACTGATGGAAGGGTTCCTGCT
BMP15	NM_001031752.1	F	TCTCAGAGGCTCCTGGCACAT
		R	TGACGAGCCCTCCTCAAGAGA
BAX	NM_173894.1	F	TTTGCTTCAGGGTTTCATC
		R	CAGCTGCGATCATCCTCT
BCL2	NM_001166486.1	F	CTGCACCTGACGCCCTTCAC
		R	GCGTCCCAGCCTCCGTTGT
KDM6B	XM_003587412.4	F	GATGACCTCTATGCTTCCAACATC
		R	CTGGTACTGATAGGCGGTGAGG
ARID2	XM_002687323.5	F	CTGGGCACAACTTCATGTGTCTG
		R	GTGCTTGTCCTGTAAGTGAGTG
SOX2	NM_001105463.2	F	GATAAGTACACACTGCCGGG
		R	CCGGGTGTTGCGGGTAG
NANOG	NM_001025344.1	F	CAGTCTCCAGCAAATGCAAG
		R	TAGAAGCCTGGGTATTCTGCC
OCT4	NM_174580.3	F	CCTGGGGGTTCTCTTTGGAA
		R	GCTTCCTCCACCCACTTCTG
DPPA3	NM_001111108.2	F	TCTCAGACTTCGCTATGCCAAAGGA
		R	CAGGTACACTGGAATCTTCGCACTC
ING3	NM_001192944.1	F	CCGCTTCCAACAACGCTT
		R	TGTCCTTCGTCCCTCTTTCATC
MSL3	NM_001192404.1	F	GCCGGAAGCCTTTCAGTCTC
		R	GAGGAACAGGCGAGGATGAT
ASF1A	NM_001076493.2	F	GTGCATCGAGGACCTGTCTG

		R	CAGGAACAGGGCCCACTAAA
ASF1B	NM_001075453.1	F	TGTCAGTATTGAACGTGGCGGTGCT
		R	TGATCTTCCACTCCAGGTCGTCTGC
